# Draft Genome Sequence of Mycobacterium parafortuitum Strain P7335

**DOI:** 10.1128/MRA.00950-18

**Published:** 2018-08-30

**Authors:** Jamal Saad, Anthony Levasseur, Michel Drancourt

**Affiliations:** a IRD MEPHI IHU Méditerranée Infection, Aix-Marseille University, Marseille, France; Georgia Institute of Technology

## Abstract

Mycobacterium parafortuitum is a rapidly growing nontuberculous mycobacterium, initially isolated from soil in Japan. The 6,175,772-bp draft genome sequence of M. parafortuitum strain P7335 exhibits a G+C content of 68.4%, 5,783 protein-coding genes, and 66 predicted RNA genes, including 59 tRNA genes, 6 rRNA operons, and 1 transfer-messenger RNA.

## ANNOUNCEMENT

The rapidly growing nontuberculous Mycobacterium parafortuitum, initially isolated from soil in Japan (1, 2), has been further isolated from water (3), nasal exudates of bovines (4), and patient sputum (5). However, M. parafortuitum is not considered a pathogenic species (6, 7). A so-called M. parafortuitum complex was initially defined on the basis of phenotypic characteristics shared by several species (5, 8) comprising Mycobacterium neoaurum (9, 10), Mycobacterium hodleri (11), Mycobacterium aurum, Mycobacterium vaccae, Mycobacterium diernhoferi, Mycobacterium austroafricanum (5), and Mycobacterium frederiksbergense (12). This classification along with the validity of some of these species was then challenged by experimental DNA-DNA hybridization studies (13). In order to further contribute to the delineation of the so-called M. parafortuitum complex, we sequenced the genome of M. parafortuitum strain P7335 after it was cultured for 7 days at 37°C on Middlebrook 7H10 agar supplemented with 10% oleic albumin dextrose catalase (OADC) (Becton, Dickinson, Sparks, USA) under a 5% CO_2_ atmosphere. DNA was extracted using InstaGene matrix (Bio-Rad, Marnes-la-Coquette, France), and 0.2 µg/µl DNA was sequenced with the MiSeq platform (Illumina, Inc., San Diego, CA, USA). Seven runs were made with read lengths of 2 × 250 bp over 39 h. Two banks were prepared in the mate-paired format, one with inserts of 13 kb and one with inserts of 5 kb, and one bank was prepared in the paired-end format. A total of 10,804,432 reads were filtered per read qualities and assembled to reach a depth of 47-fold mean coverage by using SPAdes software (14). Contigs were combined by using SSPACE (15), GapFiller (16), and manual finishing using similarity searches and synteny block detection. The M. parafortuitum strain P7335 genome contains eight scaffolds, 6,175,772 bp, and a G+C content of 68.4%. Annotation using Prokka version 1.12 (17) yielded 5,783 protein-coding genes and 66 predicted RNA genes, including 59 tRNA genes, 6 rRNA operons, and 1 transfer-messenger RNA. Analysis using MetaPhinder-2.1 (18) showed the presence of a 5,420-bp prophage sequence exhibiting 97.9% average nucleotide identity (ANI) similarity with the enterobacterial phage phiX174 (GenBank accession number CP004084); however, its PCR amplification using primers 5’-CCCGACTGCCTATGATGTTTA-3’ (forward) and 3’-CATCTCGGCAATCTCTTTCTG-5’ (reverse) designed in this study failed, illustrating the instability of this prophage. Further in silico DNA-DNA hybridization (19) with reference genomes selected according to 16S rRNA and *rpoB* gene sequence similarity (5, 20) and measured using the Genome-to-Genome Distance Calculator (GGDC) version 2.1 (21) are presented in Table 1. In addition, the estimation of the degree of genomic similarity of strains using OrthoANI software (22) yielded ANIs ranging from 99.99% to 80.54% with M. parafortuitum CCUG 20999, M. austroafricanum, M. vanbaalenii, M. gilvum, and M. vaccae and only 76.14% to 78.05% with the other species of the M. parafortuitum complex. These in silico genomic data agree with previously published experimental data (13), confirming the inconsistency of the so-called M. parafortuitum complex. As a consequence, the taxonomic positions of M. aurum, M. neoaurum, and M. diernhoferi need to be reevaluated.

**TABLE 1:** Comparison of M. parafortuitum P7335 with reference Mycobacterium species using GGDC, formula 2

Comparative genome sequence (GenBank or NCBI RefSeq no.)	Reference	DDH^*a*^ +/− SD
M. parafortuitum (MVID00000000)	23	98.4 ± 1.3
M. gilvum (NC_009338)	24	26.5 ± 4.9
M. vanbaalenii (NC_008726)		25 ± 4.8
M. vaccae (NZ_CP011491)	25	24.8 ± 4.8
M. austroafricanum (NZ_CCAW000000000)	26	24.9 ± 4.8
M. chubuense (NC_018027)	27	23.1 ± 4.8
M. obuense (NZ_JYNU00000000)	28	22.1 ± 4.7
M. rutilum (NZ_LT629971)		21.8 ± 4.7
M. diernhoferi (MPNS00000000)		20.9 ± 4.7
M. aurum (NZ_LT549889)		20.9 ± 4.6
M. rhodesiae (NC_016604)		20.5 ± 4.7
M. neoaurum (NC_023036)	29	20.3 ± 4.7
M. litorale (NZ_CP019882)		20.3 ± 4.6

a DNA-DNA hybridization (DDH) estimates are based on identities/genome lengths.

### Data availability.

The draft genome sequence of M. parafortuitum strain P7335 has been deposited at EBI/GenBank under the accession numbers UEGS01000001 to UEGS01000008 .
